# Recognizing intertwined patterns using a network of spiking pattern recognition platforms

**DOI:** 10.1038/s41598-022-23320-8

**Published:** 2022-11-14

**Authors:** Masoud Amiri, Amir Homayoun Jafari, Bahador Makkiabadi, Soheila Nazari

**Affiliations:** 1 Department of Medical Physics and Biomedical Engineering, School of Medicine, Tehran University of Medical Science (TUMS), Tehran, Iran; 2 Research Center for Biomedical Technologies and Robotics (RCBTR), Advanced Medical Technologies and Equipment Institute (AMTEI), Tehran University of Medical Science (TUMS), Tehran, Iran; 3 Faculty of Electrical Engineering, Shahid Beheshti University, Tehran, Iran

**Keywords:** Computational biology and bioinformatics, Neuroscience, Engineering

## Abstract

Artificial intelligence computing adapted from biology is a suitable platform for the development of intelligent machines by imitating the functional mechanisms of the nervous system in creating high-level activities such as learning, decision making and cognition in today's systems. Here, the concentration is on improvement the cognitive potential of artificial intelligence network with a bio-inspired structure. In this regard, four spiking pattern recognition platforms for recognizing digits and letters of EMNIST, patterns of YALE, and ORL datasets are proposed. All networks are developed based on a similar structure in the input image coding, model of neurons (pyramidal neurons and interneurons) and synapses (excitatory AMPA and inhibitory GABA currents), and learning procedure. Networks 1–4 are trained on Digits, Letters, faces of YALE and ORL, respectively, with the proposed un-supervised, spatial–temporal, and sparse spike-based learning mechanism based on the biological observation of the brain learning. When the networks have reached the highest recognition accuracy in the relevant patterns, the main goal of the article, which is to achieve high-performance pattern recognition system with higher cognitive ability, is followed. The pattern recognition network that is able to detect the combination of multiple patterns which called intertwined patterns has not been discussed yet. Therefore, by integrating four trained spiking pattern recognition platforms in one system configuration, we are able to recognize intertwined patterns. These results are presented for the first time and could be the pioneer of a new generation of pattern recognition networks with a significant ability in smart machines.

## Introduction

The nervous system consumes a very low power of 10–20 watts in cognitive processes and pattern recognition tasks^1^. Considering that in the process of designing chips, power consumption is a very important issue, the development of bio-inspired pattern recognition networks with the ability to be implemented on neuromorphic chips has attracted a lot of attention^2–6^. In recent years, the energy consumption in neuromorphic systems for the transmission of each spike has reached less than 0.02 PJ^7–11^, this is the reason why the development of AI networks with the ability to implement on such hardware have attracted much attention^4,12,13^. Therefore, we focus on the development of artificial intelligence network based on biological spiking networks with the ability to implement on normorphic hardware.

Also, in recent studies, deep learning techniques have attracted a lot of attention in the scientific community, so that deep networks have been used in many applications of supervised and reinforcement learning. Convolution neural networks (CNN) are the most popular type of deep neural networks that are able to automatically extract features from input data.

This article is an important step in introducing new learning methods based on the nervous system learning approach in bio-inspired spiking neural networks (SNN) that, in addition to being able to compete with deep learning, can be implemented on neuromorphic chips. Here we introduce biologically plausible SNNs that are trained based on the spatial-STDP (an extended version of spike time dependent plasticity), so that the transmission of AMPA and GABA neurotransmitters adjusts the synaptic weights of the spiking platforms to learn unlabeled patterns.

The common dataset used to test network performance was the MNIST dataset, but due to the fact that many studies have been done on MNIST, this database has been solved and is known as a non-challenging dataset. That's why in April 2017, an extended version of MNIST including handwritten digits and letters was released called EMNIST, which is a larger dataset with more diverse data from different sources. Until now, however, achieving high-performance accuracy in recognizing EMNIST, ORL and YALE datasets using spiking pattern recognition networks remains a challenge that will be addressed in this article.

Nevertheless, four pattern recognition networks, which are called pattern recognition networks based on SNN1, SNN2, SNN3, and SNN4 were trained based on spatial-STDP on the digits of EMNIST, letters of EMNIST, YALE and ORL faces, respectively. Finally, digit, letter, YALE, and ORL recognition networks can detect digits and letters of EMNIST, YALE, and ORL faces with an accuracy of 97.8%, 93.5%, 97.2%, and 99.5%, respectively.

Several pattern recognition networks have been developed that identify patterns of one dataset with an acceptable accuracy but until now, a pattern recognition network is not provided that can detect the patterns, which are created by combinations of two or three datasets. The important goal that follows in this paper is to make a network of pattern recognition platforms based on SNN1, SNN2, SNN3, and SNN4 for integrating the ability of them in one system in order to recognize the patterns, which are created by the combination of two or three datasets (called intertwined patterns). So far, no spiking network has been introduced for recognizing the intertwined patterns. Therefore, the results of this article can be the leading study in the new generation spiking pattern recognition networks for identifying the complex patterns.

All simulations have been performed with the Runge–Kutta algorithm at time step $\Delta t=0.05$ ms. All procedures were configured with c +  + language by the Visual Studio software.

In the next section, we explain the method including the neuron and synapse model, network architecture, training, and classification method. Sections III & IV contain performance evaluation of the designed pattern recognition networks and the networking of spiking pattern recognition platforms based on SNN1, SNN2, SNN3, and SNN4 to identify the intertwined patterns and finally, section V concludes the paper.

## Architecture and learning mechanism of the pattern recognition network based on SNN1 (digit recognition)

The structure of the digit recognition network consists of three parts. The input layer transmits information of image to a population consisting of thousands of excitatory and inhibitory neurons that exchange information through the transmission of AMPA and GABA neurotransmitters. By receiving the spikes of the neural population, the neurons of the output layer are responsible for classifying the images. In the following, different parts of the network will be discussed.

### Neuron and synapse model

The considered neurons model in digit recognition network is leaky integrate and fire (LIF) neurons. ${v}_{k}(t)$ is defined as the membrane potential of a neuron as follows:

1 $${\tau }_{m}\frac{d{v}_{k}}{dt}=-{v}_{k}(t)+{I}_{AK}(t)-{I}_{GK}(t)$$

For each neuron, excitatory synaptic current (${I}_{AK}$) and inhibitory synaptic current (${I}_{GK}$) are defined, which are modeled based on the following equations.

2 $${\tau }_{dA}\frac{{dI}_{Ak}}{dt}=-{I}_{Ak}+{x}_{Ak}$$

3 $${\tau }_{rA}\frac{{dx}_{AK}}{dt}={-x}_{Ak}+{\tau }_{m}\left({J}_{k-Pyr}\sum_{pyr}\delta (t-{t}_{k-pyr}-{\tau }_{L})+{J}_{k-ext}\sum_{ext}\delta (t-{t}_{k-ext}-{\tau }_{L})\right)$$

4 $${\tau }_{dG}\frac{{dI}_{Gk}}{dt}=-{I}_{Gk}+{x}_{Gk}$$

5 $${\tau }_{rG}\frac{{dx}_{AG}}{dt}={-x}_{AG}+{\tau }_{m}\left({J}_{k-int}\sum_{int}\delta (t-{t}_{k-int}-{\tau }_{L})\right)$$

where ${\tau }_{m}$ (20 and 10 ms for excitatory and inhibitory neurons), ${v}_{thr}$ (18 mv), ${v}_{res}$ (0 mv), ${\tau }_{rp}$ (2 and 1 ms for excitatory and inhibitory neurons), and ${\tau }_{L}(1$ ms) respectively are defined as membrane time constant, threshold of neurons firing, resting potential, refractory time and Latency of the post-synaptic currents^13,14^. Also, ${t}_{k-pyr,int,ext}$, ${\tau }_{dA}({\tau }_{dG})$ and ${\tau }_{rA}({\tau }_{rG})$ respectively describe time of received spike from pyramidal neurons/interneurons/external inputs to neuron *k,* decay time and rise time of the excitatory (inhibitory) AMPA (GABA) synaptic currents^14^. Excitatory and inhibitory synaptic weights play a major role in network learning which is presented by ${J}_{k-pyr}$ (excitatory synapse from pyramidal neuron to neuron *k*), ${J}_{k-int}$ (inhibitory synapse from inter-neuron to neuron *k*), and ${J}_{k-ext}$ (excitatory synapse from input to neuron *k*).

### Architecture of digit recognition network

#### Input layer

EMNIST dataset contains 240,000 training examples and 40,000 test examples of digits 0 to 9, which patterns are 128*128 pixels. Previous studies^15–18^ have shown that it is better to consider a one-to-one correspondence between the number of the pixels of the input pattern and the number of neurons in the input layer. Obviously, as the number of pixels of input pattern increases, the number of input nodes increases dramatically. In this paper, the image is mapped to a sinusoidal signal that makes the pattern recognition network independent of the image size. The mapping of image is created by translating the vertical and horizontal position of the pixel into the frequency and phase of the sine signal with the amplitude proportional to $pixel brightness+constant$. Unlike previous work^19,20^, the brightness intensity of the image pixels is summed with a constant value so that the information of the black pixels is not removed.

Finally, signal representing the image is defined as follows:

6 $$\mathrm{signal \, representing \, the \, image}=\sum_{i=1}^{N}\sum_{j=1}^{M}{(pixel \, britnes}_{ij}+constant)\mathrm{sin}({F}_{i,j}*t+{\varphi }_{i,j})$$

where $i$,$j$ indicates the number of rows and columns of image and ${F}_{i,j}$ and ${\varphi }_{i,j}$ are defined as follows^19^:

7 $${F}_{i,j}=j*\frac{{F}_{max}}{M} \& {\varphi }_{i,j}=i*\frac{360}{N}$$

In fact, the presented coding is an efficient dimensionality reduction method that, unlike PCA, where part of the information is removed, there is a one-to-one correspondence between the obtained signal and the image. Therefore, it is possible to return from the signal to the image without losing information, and this is the strength of this coding.

The input node produces Poisson spike train with a rate that varies over time and in proportion to the amplitude of the signal representing image. This spike train feed the neural population with image information through excitatory synapses. It means that the time-varying rate of Poisson spike train with the mean of the obtained signal of image inject to the second layer. Each neuron in the second layer receives an external excitatory input.

#### Second layer

**Figure 1:**
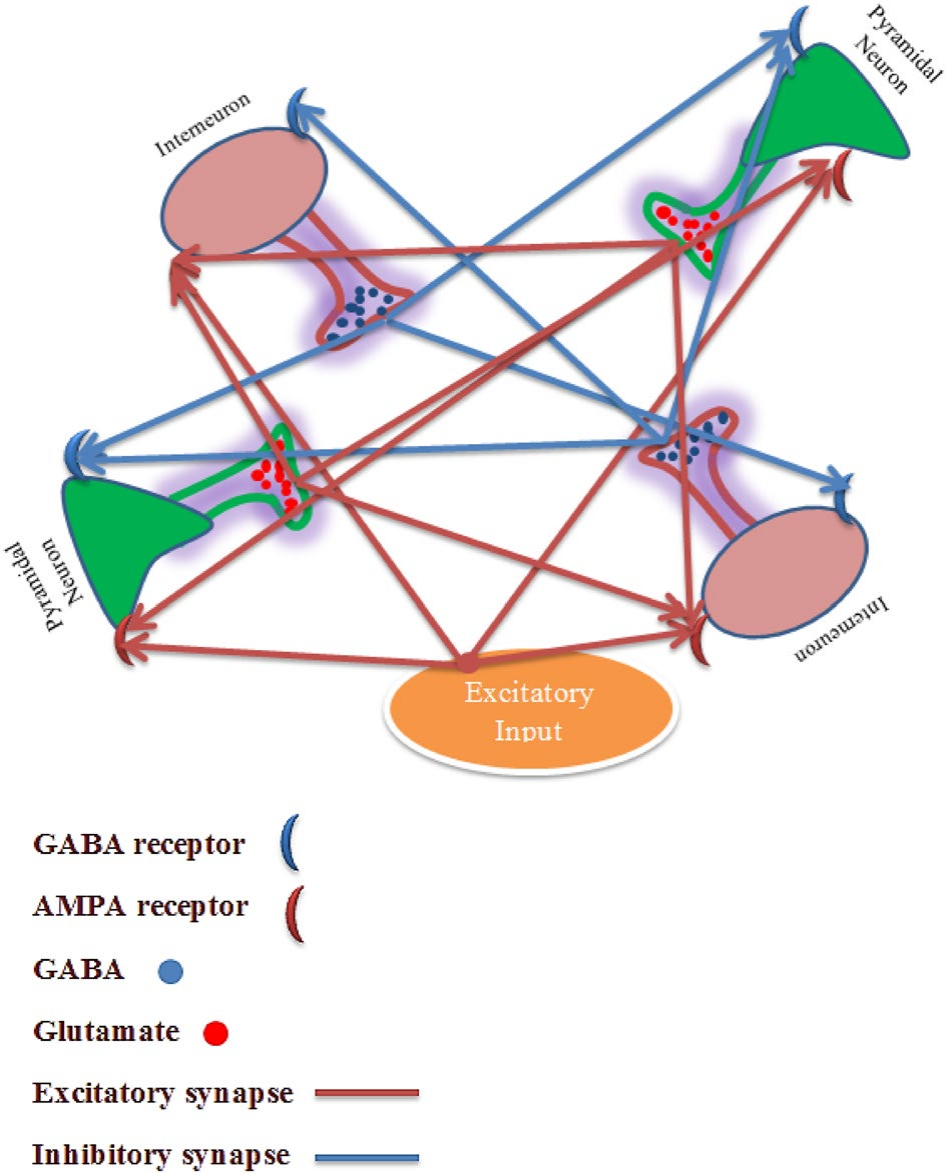
As can be seen, pyramidal neurons send excitatory signals to connected neurons via excitatory neurotransmitters and interneurons send the inhibitory signal to connected neurons via inhibitory neurotransmitters.

The neural population as second layer of digit recognition network contains 5000 LIF neurons which 80% of them^21,22^ are excitatory neuron called pyramidal neuron and the remaining are inhibitory neurons called interneurons. Due to the direct role of synapses in network learning, the dynamics of synapses originates from the biological reality of learning in the nervous system, so that the excitatory and inhibitory AMPA and GABA neurotransmitters play the role of excitatory and inhibitory synapses. Pyramidal neurons excite connected neurons, while inhibitory neurons inhibit connected neurons. The neural interactions in a small population of excitatory and inhibitory neurons are shown in Fig. 1.

**Figure 2:**
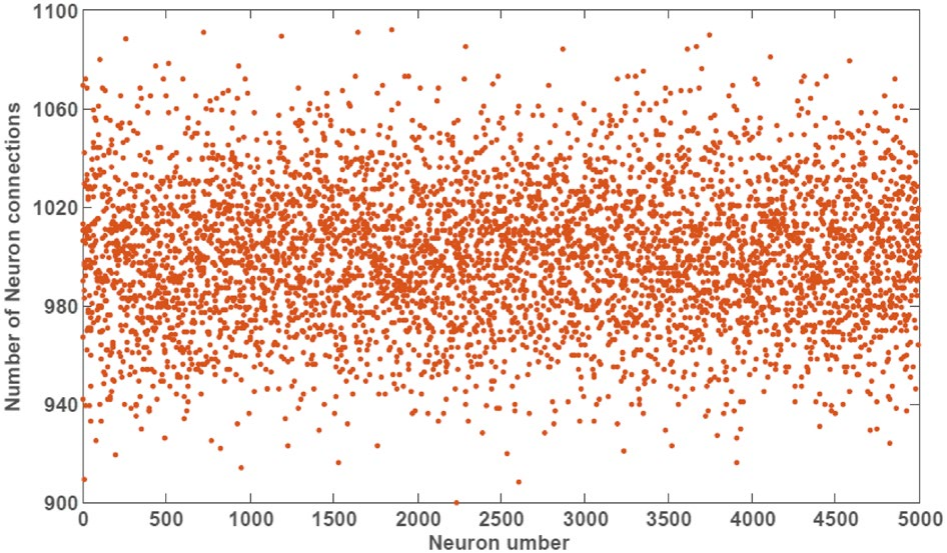
Number of excitatory and inhibitory synapses (connections) for each neuron of digit recognition network. Each point in the figures indicates number of connections (synapses) for the corresponding neuron number on the x-axis.

In addition to the importance of the dynamics of excitatory and inhibitory synapses in the spiking network, the number of network synapses will affect the learning power. Due to the importance of implementing pattern recognition networks on neuromorphic boards, network with fully connections imposes high power consumption, implementation complexity and speed limitation on hardware^9,23^. On the other hand, some studies have shown that by reducing fully connected network connections by up to 90% and creating a sparsely-connected network, performance accuracy can be improved^24^. Also, based on the biological findings, generally the connection probability between two neurons in the nervous system is 0.2^25,26^. Therefore, over 5 million (exactly 5,001,325) excitatory and inhibitory synapses are considered in the second layer of digit recognition network. Figure 2 shows the number of synapses for each neuron in the second layer of digit recognition network.

In order to consider the spatial distribution of the learning process, the neurons are arranged in a rectangular space with 100*50 and 100*70 nodes, respectively. The interaction between the connected neurons decreases exponentially, i.e. neurons that are farther apart have less effect on each other. Therefore, by adding ${e}^{\frac{-r}{D}}$ in Eqs. 3 and 5, the decreasing effect of neurons on each other by increasing the distance has been considered in the dynamical equations of the excitatory and inhibitory synapses.

8 $${\tau }_{rA}\frac{{dx}_{AK}}{dt}={-x}_{Ak}+{\tau }_{m}\left({e}^{\frac{-r}{D}}{J}_{k-Pyr}\sum_{pyr}\delta (t-{t}_{k-pyr}-{\tau }_{L})+{J}_{k-ext}\sum_{ext}\delta (t-{t}_{k-ext}-{\tau }_{L})\right)$$

9 $${\tau }_{rG}\frac{{dx}_{AG}}{dt}={-x}_{AG}+{{e}^{\frac{-r}{D}}\tau }_{m}\left({J}_{k-int}\sum_{int}\delta (t-{t}_{k-int}-{\tau }_{L})\right)$$

where *r* in Eq. (8) denotes the distance between pyramidal neuron and neuron *k* and *r* in Eq. (9) indicates the distance between interneuron and neuron *k*. Also, *D* is the scaling parameter of the distance between neurons.

#### Output layer

**Figure 3:**
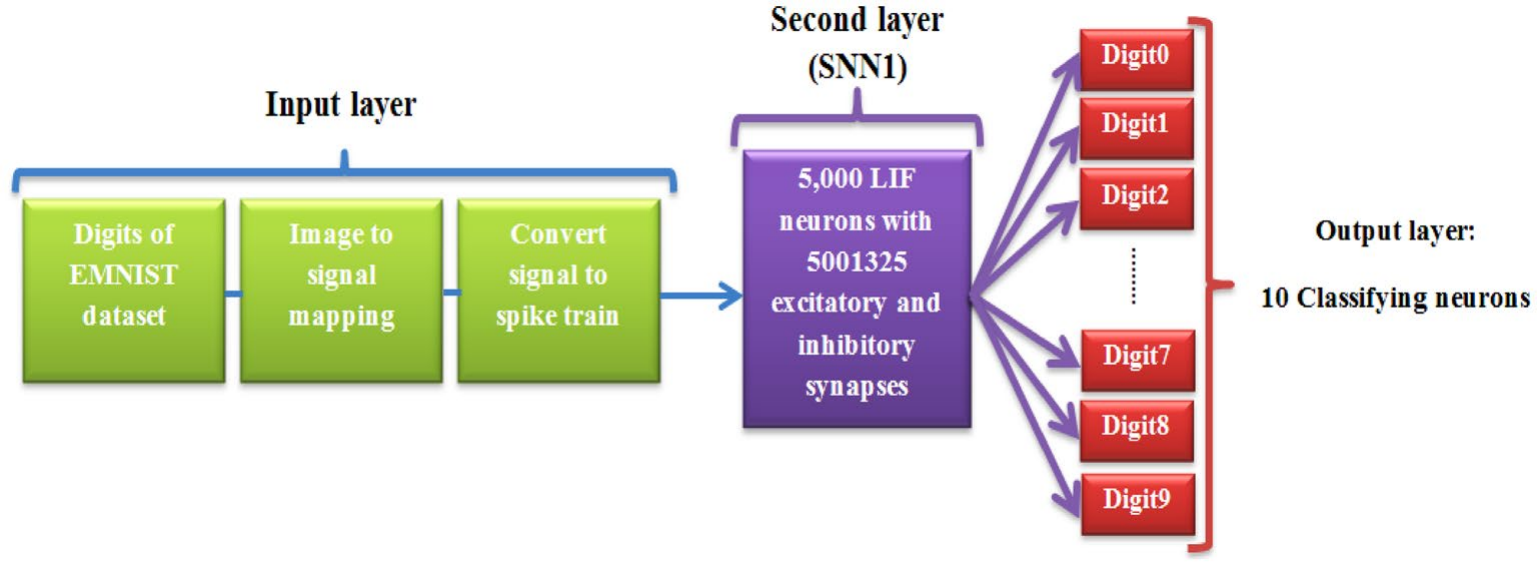
The general schematic of the pattern recognition network based on SNN1 (digit recognition network).

According to the number of dataset classes, the LIF neurons are placed in the output layer of the pattern recognition network. Therefore, output layer of digit recognition network consists of 10 LIF neurons which have full connections to all neurons of second layer. Output layer neurons called classifying neurons receive their excitatory and inhibitory input from pyramidal neurons and interneurons, respectively. Nevertheless, the general architecture of the digit recognition network (pattern recognition network based on SNN1) is shown in Fig. 3.

In the topology of the spiking network, instead of using a complex neuron model such as the Hodgkin-Huxley neuronal model, the LIF neuron model can be used to enable simulating the network in large dimensions. On the other hand, the proposed spiking pattern recognition network is modeled based on complicate neural interactions. The neural interactions based on transmission of excitatory and inhibitory neurotransmitters, AMP and GABA play an important role in learning process. In the next section, the learning process is fully described.

### Learning

There is no doubt that artificial intelligence has gone far beyond the human nervous system in many aspects of computing. But to date, artificial intelligence is much weaker than the brain in terms of cognitive aspects such as learning, decision-making and generalization power. Therefore, imitating the function of the nervous system in learning can be an idea to improve the cognitive ability of smart machines. For this reason, spiking neural networks and spike-based learning methods have received much attention from researchers today^27–29^. In spiking networks, spike neurons exchange information through synapses. Usually, temporally information coding is considered in the training procedure of spiking networks, while the spatial feature of learning in the nervous system is of considerable importance, which is why spatial–temporal learning is introduced in this article. Accordingly, a new version of the spike-timing-dependent plasticity mechanism has introduced called “spatial spike-timing-dependent plasticity (Spatial-STDP)”, so that the online allocation of synaptic weights is based on the temporal and spatial information of the released spikes from neurons. In addition to this innovation, we tried to enhance the learning process through Spatial-STDP and adapt it to the principles of learning in the nervous system. In this regard, based on biological observation, Spatial-STDP affects the transmissions of AMPA and GABA neurotransmitters during the learning process. Similar to Hebbian learning^30,31^, STDP and spatial-STDP are learning approach based on biological evidence of nervous system learning. In fact, in spatial-STDP learning procedure, the synaptic weights are updated based on the spike activity of the presynaptic and postsynaptic neurons and the distance between them, and this process continues until the network learn the patterns.

In the spatial-STDP learning, the synaptic weight between presynaptic neuron *i* and postsynaptic neuron *j* can be updated as follows^32^.

10 $$\Delta {w}_{j}=\sum_{k=1}^{N}{\sum }_{l=1}^{N}W({t}_{j}^{l}-{t}_{i}^{k})$$

${l}{th}$, ${k}{th}$ spike of postsynaptic and presynaptic neuron are denoted by ${t}_{j}^{l}$, ${t}_{i}^{k}$, respectively. $W\left(x\right)$ as a function of changing synaptic weight based on the time of spikes of pre- and post-synaptic neurons can be defined as follows:

11 $$W\left(x\right)=\left\{\begin{array}{c}{{e}^\frac{r}{Ds}A}_{+}\mathrm{exp}\left(\frac{-x}{{\tau }_{+}}\right) if x>0\\ {{e}^\frac{r}{Ds}A}_{-}\mathrm{exp}\left(\frac{x}{{\tau }_{-}}\right) otherwise\end{array}\right.$$

${\tau }_{+}$ and ${\tau }_{-}$ indicate time constants of exponentially increase or decrease in synaptic weight and *A* is constant parameter as learning rate. The spatial feature has been added to Eq. (11) with parameter *r* which is expresses the distance between two neurons, and $Ds$ is a constant parameter of distance scale. In fact, terms $\mathrm{exp}\left(\frac{-x}{{\tau }_{+}}\right)$ and ${e}^\frac{r}{Ds}$ represent the temporal and spatial features of learning, respectively.The spatial term of learning increases the speed of the training convergence. Acceleration of learning occurs for the following two reasons:

1. If two neurons are far apart and their spike intervals are short, more weight increasing will occur between them than STDP.
2. Conversely, if two neurons are close together and their spike intervals are short, less weight increasing will occur between them than STDP

**Figure 4:**
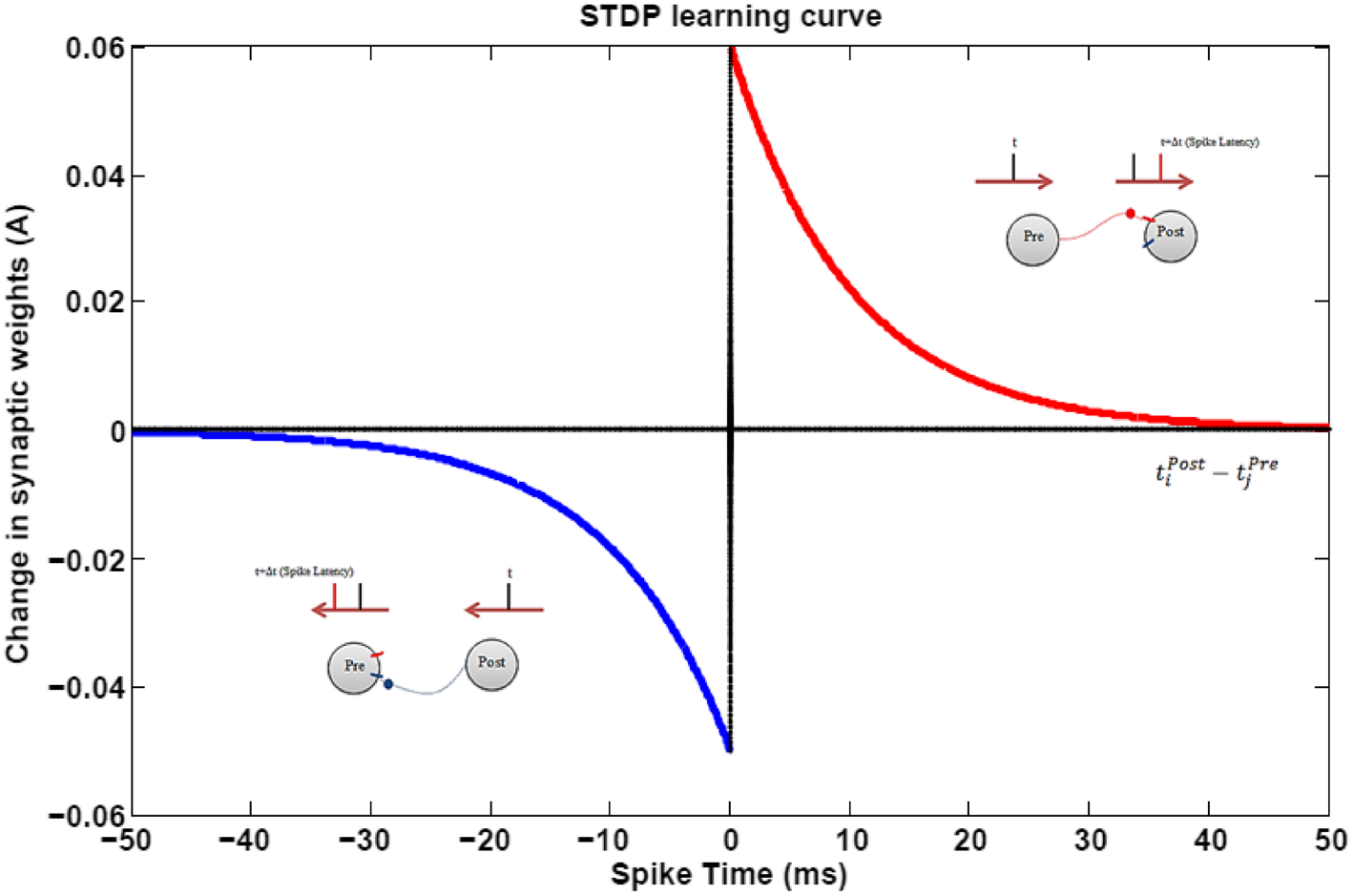
LTP ($t_{i}^{post} - t_{j}^{pre} > 0$) and LTD ($t_{i}^{post} - t_{j}^{pre} < 0$) learning rule in the spike-time-dependent-plastisity model.

In Fig. 4, considering the constant spatial distance, the synaptic weight changes are plotted as a function of the spike time generated by the pre- and post-synaptic neurons.

Figure 4 shows that if the presynaptic neuron fires before the postsynaptic neuron, the synaptic weight between them increases (long-term potentiation (LTP)) according to Eq. (11), and conversely, if the postsynaptic neuron fires before the presynaptic neuron, the synaptic weight between them reduces (long-term depression (LTD)). In fact, what is evident is that the spike times of the pre- and post-synaptic neurons play the most important role in synaptic weight change (synaptic plasticity)^32^. It is important to note that in Fig. 4, the distance between the pre- and post-synaptic neurons is considered to be a unit, and it is obvious that by increasing the distance according to the Eq. (11), the rate of increase and decrease of synaptic weight will change.

Comprehension of cellular processes and the principles of dynamics governing nervous system interactions is very difficult due to the diversity of biological cells and the complexity and diversity of information-transmitting neural synapses. In order to find a relative understanding of the mechanism of synaptic weight change and synapse plasticity in the learning process, tracking and discovering the structure and functional behavior of ion channels and neurotransmitters can be useful. One of the most important biological aspects of learning in the nervous system is the discovery of the effective effect of AMPA (excitatory) and GABA (inhibitory) neurotransmitters on the regulation of synaptic weight change and finally synaptic plasticity^33–35^. On the other hands, spike-timing-dependent plasticity (STDP) is a biological process that regulates the strength of connections between neurons in the nervous system^36^. In this article, these two biological findings have been integrated and STDP learning is defined based on the transmission of AMPA and GABA neurotransmitters. Also, another biological finding shows that as the distance between neurons increases, their interaction through synapse decreases^37^. Therefore, we have applied this biological finding on the learning model of STDP based on AMPA and GABA transmissions and introduce spatial-STDP learning. In spatial-STDP in addition to synaptic change based on spike times, with increasing distance the interaction between the connected neurons decreases exponentially, i.e. neurons that are farther apart have less effect on each other. In general, spatial-STDP learning is created by combining three proven biological principles.

The transmission of AMPA neurotransmitters in the synaptic space has been proven to play an important role in information storage and learning in the nervous system^33^. In fact, increasing or decreasing the AMAP neurotransmitters in the synaptic space causes LTP and LTD in the learning process^33^. Also, the increase and decrease of GABA neurotransmitters have a significant effect on the change of synaptic weights, and as a result, LTD and LTP which finally lead to the learning process^34,35^. Based on scientific published studies, the occurrence of the learning process or in other words synaptic weight changes based on LTP and LTD is influenced by the transmission of the AMPA and GABA neurotransmitters in synaptic space. In this regard, we tried to combine artificial intelligence with biological findings to enhance the level of learning. Therefore, the spatial-STDP learning approach is defined on the dynamic equations of AMPA and GABA currents so that we can simulate learning processes LTP and LTD by adjusting the transmission of AMPA and GABA neurotransmitters.

As denoted, adjustment of neurotransmitters transmissions such as AMPA and GABA is a fundamental mechanism for the synapse plasticity, which is the basis of the learning method in the present paper. Therefore, LTP process of learning defined based on the following rules.

1. In the connection of pyramidal neuron to pyramidal neuron with the random initial weight based on Gaussian random distribution with a mean of zero and a standard deviation of 0.2, if post-synaptic neuron fires after presynaptic-neuron, then the AMPA synaptic weight between them increases with the size of ${e}^\frac{r}{Ds}*0.06*\mathrm{exp}(-\frac{\left({t}_{post-pyr}-{t}_{pre-pyr}\right)}{10})$ according to Eq. (12).
2. In the connection of interneuron to pyramidal neuron with the random initial weight based on Gaussian random distribution with a mean of zero and a standard deviation of 0.2, if post-synaptic neuron fires after presynaptic-neuron, then the GABA synaptic weight between them decreases with the size of $-{e}^\frac{r}{Ds}*0.05*\mathrm{exp}(-\frac{\left({t}_{post-pyr}-{t}_{pre-int}\right)}{10})$ according to Eq. (13).
3. In the connection of pyramidal neuron to interneuron with the random initial weight based on Gaussian random distribution with a mean of zero and a standard deviation of 0.2, if post-synaptic neuron fires after presynaptic-neuron, then the AMPA synaptic weight between them increases with the size of ${e}^\frac{r}{Ds}*0.06*0.4*\mathrm{exp}(-\frac{\left({t}_{post-int}-{t}_{pre-pyr}\right)}{10})$ according to Eq. (14).
4. In the connection of interneuron to interneuron with the random initial weight based on Gaussian random distribution with a mean of zero and a standard deviation of 0.2, if post-synaptic neuron fires after presynaptic-neuron, then the GABA synaptic weight between them decreases with the size of $-{e}^\frac{r}{Ds}*0.05*0.4*\mathrm{exp}(-\frac{\left({t}_{post-int}-{t}_{pre-int}\right)}{10})$ according to Eq. (15).

In the case of LTD (post-synaptic neuron fires before presynaptic-neuron) based on the Fig. 4, weight change is reversed.

The cause of the displacement of $\sum_{Pyr}\dots$ in Eqs. (5), (12) is that when the network is training, the connecting weights of all pyramidal neurons to neuron *k* are not equal so that each pyramidal neuron is attached to neuron *k* with its own weight. Therefore, the efficacy of connections ${J}_{k-Pyr}+({e}^{\frac{-r}{D}}A\mathrm{exp}(-\frac{\left({t}_{k}-{t}_{pyr}\right)}{{\tau }_{+}}))$ was multiplied by the Dirac function and then summed.

The following equations (Eqs. 12–13 for pyramidal neurons and Eqs. 14–15 for interneurons) show the equations governing the topology and learning of pattern recognition network.

12 $$ \begin{aligned}{\tau }_{rA}\frac{{dx}_{AK}}{dt}&={-x}_{Ak}+{\tau }_{m}\left({e}^{\frac{-r}{D}}\left({J}_{k-Pyr}+\sum_{k=1}^{N}{\sum }_{l=1}^{N}\left({e}^\frac{r}{Ds}A\mathrm{exp}\left(-\frac{\left({t}_{j}^{l}-{t}_{i}^{k}\right)}{{\tau }_{+}}\right)\right)\right)\right. \\&\quad \left.\sum_{pyr}\delta (t-{t}_{k-pyr}-{\tau }_{L})+{J}_{k-ext}\sum_{ext}\delta (t-{t}_{k-ext}-{\tau }_{L})\vphantom{\left({e}^{\frac{-r}{D}}\left({J}_{k-Pyr}+\sum_{k=1}^{N}{\sum }_{l=1}^{N}\left({e}^\frac{r}{Ds}A\mathrm{exp}\left(-\frac{\left({t}_{j}^{l}-{t}_{i}^{k}\right)}{{\tau }_{+}}\right)\right)\right)\right.}\right) A=\left\{\begin{array}{c}{A}_{+} if {t}_{j}^{l}-{t}_{i}^{k}>0\\ {A}_{-} if {t}_{j}^{l}-{t}_{i}^{k}<0\end{array}\right.\end{aligned} $$

13 $$ \begin{aligned}{\tau }_{rG}\frac{{dx}_{AG}}{dt}&={-x}_{AG}+{\tau }_{m}\left({e}^{\frac{-r}{D}}\left({J}_{k-int}+\sum_{k=1}^{N}{\sum }_{l=1}^{N}\left({e}^\frac{r}{Ds}A\mathrm{exp}\left(\frac{\left({t}_{j}^{l}-{t}_{i}^{k}\right)}{{\tau }_{-}}\right)\right)\right)\right.\\&\quad\left.\sum_{int}\delta (t-{t}_{k-int}-{\tau }_{L})\vphantom{\left({e}^{\frac{-r}{D}}\left({J}_{k-int}+\sum_{k=1}^{N}{\sum }_{l=1}^{N}\left({e}^\frac{r}{Ds}A\mathrm{exp}\left(\frac{\left({t}_{j}^{l}-{t}_{i}^{k}\right)}{{\tau }_{-}}\right)\right)\right)\right.}\right) A=\left\{\begin{array}{c}{A}_{-} if {t}_{j}^{l}-{t}_{i}^{k}>0\\ {A}_{+} if {t}_{j}^{l}-{t}_{i}^{k}<0\end{array}\right.\end{aligned} $$

14 $$ \begin{aligned} \tau_{rA} \frac{{dx_{AK} }}{dt} & = - x_{Ak} + \tau_{m} \left( {e^{{\frac{ - r}{D}}} \left( {J_{k - Pyr} + \mathop \sum \limits_{k = 1}^{N} \mathop \sum \limits_{l = 1}^{N} \left( {e^{\frac{r}{Ds}} B{\text{exp}}\left( { - \frac{{\left( {t_{j}^{l} - t_{i}^{k} } \right)}}{{\tau_{ + } }}} \right)} \right)} \right)}\right.\\ & \quad\left. {\mathop \sum \limits_{pyr} \delta (t - t_{k - pyr} - \tau_{L} ) + J_{k - ext} \mathop \sum \limits_{ext} \delta (t - t_{k - ext} - \tau_{L} )} \vphantom{\left( {e^{{\frac{ - r}{D}}} \left( {J_{k - Pyr} + \mathop \sum \limits_{k = 1}^{N} \mathop \sum \limits_{l = 1}^{N} \left( {e^{\frac{r}{Ds}} B{\text{exp}}\left( { - \frac{{\left( {t_{j}^{l} - t_{i}^{k} } \right)}}{{\tau_{ + } }}} \right)} \right)} \right)}\right.}\right) B = \left\{ {\begin{array}{*{20}c} {B_{ + } if t_{j}^{l} - t_{i}^{k} > 0} \\ {B_{ - } if t_{j}^{l} - t_{i}^{k} < 0} \\ \end{array} } \right. \end{aligned} $$

15 $$ \tau_{rG} \frac{{dx_{AG} }}{dt} = - x_{AG} + \tau_{m} \left( {e^{{\frac{ - r}{D}}} \left( {J_{k - int} + \mathop \sum \limits_{k = 1}^{N} \mathop \sum \limits_{l = 1}^{N} \left( {e^{\frac{r}{Ds}} B{\text{exp}}\left( {\frac{{\left( {t_{j}^{l} - t_{i}^{k} } \right)}}{{\tau_{ - } }}} \right)} \right)} \right)\mathop \sum \limits_{int} \delta (t - t_{k - int} - \tau_{L} )} \right) \quad \quad B = \left\{ {\begin{array}{*{20}c} {B_{ - } if t_{j}^{l} - t_{i}^{k} > 0} \\ {B_{ + } if t_{j}^{l} - t_{i}^{k} < 0} \\ \end{array} } \right. $$

The learning process is such that if the presynaptic neurons spike before the pyramidal neurons, according to the distance criterion between two neurons, the injection of AMPA and GABA neurotransmitters into the synaptic space increases and decreases, respectively. Also, if the presynaptic neurons spike before the interneuron, according to the distance criterion between two neurons, the injection of AMPA and GABA neurotransmitters into the synaptic space decreases and increases, respectively. As the rate of the spikes in the pattern recognition network increases, so does the speed of learning i.e. interneurons release fewer neurotransmitters into the synaptic cleft than pyramidal neurons^38–40^.

### Training and classification

The introduced network is trained un-supervised on all training set of digits of EMNIST, four times, so that 3 training epochs considered for learning and last epoch is considered to assigning each of the classifying neurons to one class based on the maximum firing rate. During network training, all weights except the input weights of the network are trained based on the proposed learning approach. Between two images applied to the pattern recognition network, we create an interval of 100 ms until the dynamic equations reach their stable state. After the completion of the training epochs, in the testing phase, the synaptic weights are considered fixed and do not change. The criterion in evaluating the performance of the network is the correct recognition rate of test-set based on the maximum firing rate of the output layer neurons. This means that pattern recognition network classify correctly when the firing rate of class-assigned output neuron is higher than that of other output layer neurons.

### The evaluation of classification accuracy

After completing the learning steps, digit recognition network using adjusted synaptic weights through spatial-STDP can identify test samples belong to which group of output classes. The digit recognition network is trained and tested with 4000 pyramidal neurons, 1000 interneurons, and 10 output classifying neurons using all samples of the EMNIST digits dataset. In the learning phase, learnable synaptic weight are changing and updating to learn training samples, while in the testing phase, the performance of pattern recognition network is evaluated with the fixed structure and synaptic weights.

In this section, the performance and accuracy of the digit recognition network is evaluated based on the correct classification of all test data. Based on the high accuracy of the obtained results, the approach considered in adapting machine learning to nervous system learning is based on sound reasoning. A noteworthy point in the results that is important in the implementation of low-consumption pattern recognition networks with online learning capability on the hardware is that network neurons have sparse spike activity. Also, the classifying neurons (output layer neurons) show spike activity based on the similarity of the input pattern to the pattern they are responsible for classifying it. For example, if the input pattern is digit 1, the classifying neuron of digit 7 shows more spike activity than the digit 3 classifying neuron.

In Table 1, a summary of the latest neural networks in the recognition of digits of EMNIST is compared. The highest accuracies in the recognition of EMNIST digits have been reported by deep and convolutional networks with supervised training. According to Table 1, the proposed spatial-STDP learning approach, although is un-supervised, has the ability to compete with the today's supervised deep learning methods. In addition to the advantage of high classification accuracy, unsupervised training, the biological background of the proposed pattern recognition network can be useful as a tool in exploring the cognitive problems of the nervous system.

**Table 1:** The performance of the best published studies in recognition digits of EMNIST.

Method	Architecture	(Un-)supervised	Performance on digits
DNN	Committee of 7 CNNs^42^	Supervised	99.19%
	EDEN^43^	Supervised	99.3%
	CNN (flat; 2 conv + 1 dense)^44^	Supervised	99.46%
	Parallelized CNN^45^ Committees of neuroevolved CNNs^46^ Markov random field CNN^47^ Text Caps^48^ CNN (Spinal FC)^49^	Supervised Supervised Supervised Supervised Supervised	99.62% 99.77% 99.75% 99.79% 99.07% with 8 training epochs
	VGG-5 (Spinal FC)^49^	Supervised	99.75% with 50 training epochs
SNN	SNN using SpykeFlow^50^	Supervised	85.47% on all samples with 25 training epochs
	Proposed network	Unsupervised	97.8% with 4 training epochs

The results reported in Table 1 indicate that digit recognition network presents good performance compared to the best published studies in recent years, which confirms the correctness of the hypotheses considered in this article. In comparing the results, we tried to evaluate our work with a variety of deep and spiking networks. Also, in this article, the generalizability of digits recognition network has been considered so that, in many studies, the sharing of test and training data increases the reported accuracy and on the other hand limits the generalizability of the network^41^, which is well considered in this article by completely separating the test and training data. Evidence of high generalization ability of the digit recognition network is that in addition to separating training and test data, the training error (0.8% in Digits of EMNIST) is equal to or close to the test error (2.2% in Digits of EMNIST).

In addition to the reported results in Table 1, some related studies are introduced in^51^.

## Pattern recognition networks based on SNN2, SNN3, and SNN4

**Figure 5:**
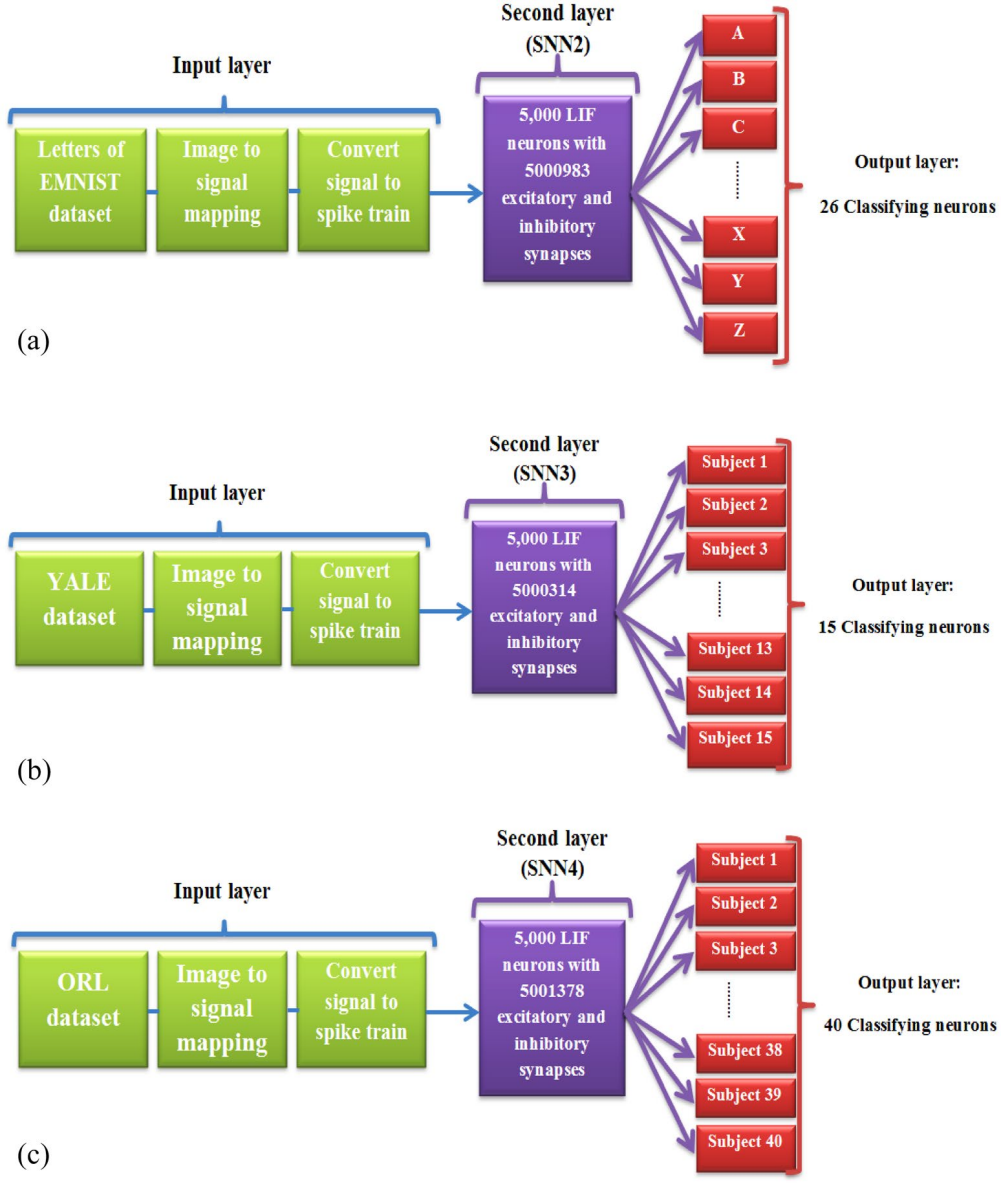
(**a**)–(**c**) are the general schematic of the proposed configurations as the pattern recognition platforms based on SNN2, SNN3, and SNN4.

In this section, three spiking pattern recognition networks with the similar structure and learning approach to pattern recognition network based on SNN1 (digit recognition network) have presented, so that pattern recognition networks based on SNN2, SNN3, and SNN4 have been trained on the handwritten letters of EMNIST, YALE faces, and ORL dataset, respectively. Therefore, four pattern recognition networks based on SNN1, SNN2, SNN3, and SNN4 have the similar structure and training method, which have trained on the four distinct datasets. The structure of pattern recognition platforms based on SNN2, SNN3, and SNN4 consist of three parts: input layer, population of 5,000 neurons and over 5 million (exactly 5,000,983, 5,000,314, and 5,001,378 in SNN2, SNN3, and SNN4, respectively) synapses as a second layer and output layer (26, 15, and 40 classifying neurons). The general structure of these pattern recognition networks is presented in Fig. 5.

Using the proposed learning approach in 2.3, the pattern recognition networks based on SNN2, SNN3, and SNN4 were trained on letters of EMNIST, YALE faces and ORL datasets, respectively. In the following, the recognition accuracy of the pattern recognition networks based on SNN2, SNN3, and SNN4 has evaluated to emphasize that the unsupervised spatial-STDP learning method indicates the best performance on other datasets.

The letters of EMNIST dataset contains 88,800 training examples and 14,800 test examples of the letters A to Z which patterns are 128*128 pixels. Classification accuracy of the pattern recognition network based on SNN2 is comparable to recent published supervised deep networks on EMNIST. Table 2 summarizes the recognition accuracy of the pattern recognition network based on SNN2 compared to recent published studies. A brief comparison confirms that proposed bio-inspired pattern recognition network based on SNN2 can compete with the previous state-of-the-art pattern recognition platforms on the letters of EMNIST dataset although it is trained unsupervised.

**Table 2:** Comparison of the classification accuracy in the letters of EMNIST dataset.

Method	Architecture	(Un-)supervised	Performance on letters
DNN	Committee of 7 CNNs^42^	Supervised	92.42%
	EDEN^43^	Supervised	88.3%
	CNN (flat; 2 conv + 1 dense)^44^	Supervised	93.63%
	Parallelized CNN^45^ Committees of neuroevolved CNNs^46^ Markov random field CNN^47^ Text Caps^48^ CNN (Spinal FC)^49^	Supervised Supervised Supervised Supervised Supervised	– 95.35% 95.44% 95.36% 90.02% with 8 training epochs
	VGG-5 (Spinal FC)^49^	Supervised	95.79% with 200 training epochs
SNN	SNN using SpykeFlow^50^	Supervised	85.47% on all samples with 25 training epochs
	Proposed network	Unsupervised	93.5% with 4 training epochs

The number of training epochs has been reported to Tables 1 and 2. As it is evident from the results of these tables, the spiking pattern recognition networks based on SNN1 and SNN2 have reached higher classification accuracy with less number of training epochs compared to previous spiking networks. Also, the spiking pattern recognition networks based on SNN1 and SNN2 have comparable accuracy to deep networks and less number of training epochs which confirms the higher convergence speed of the proposed networks in training. Therefor convergence speed of the proposed learning approach is higher than previous studies.

Considering that the number of neurons in the second layer of all spiking pattern recognition networks is considered to be 5000 neurons, in Table 3 a comparison of the relationship between the number of neurons in the second layer and the classification accuracy of EMNIST is reported.

**Table 3:** Comparison of classification accuracy in dataset MNIST by varying the number of neurons in the second layer.

Number of neurons In second layer	PY: 2000 IN: 500 All: 2500	PY: 3200 IN: 800 All: 4000	PY: 4000 IN: 1000 All: 5000	PY: 6000 IN: 1500 All: 7500
Classification accuracy on digits	90%	96.1%	97.8%	98%
Classification accuracy on letters	84.3%	91.6%	93.5%	93.9%

As it is evident from the results of the Table 3, by increasing the number of neurons more than 5000, no significant increase in classification accuracy is observed. But by reducing the number of neurons less than 5000 neurons, a significant decrease in classification accuracy is observed.

Due to the real-world applications of face recognition, the creation of artificial intelligence networks for this purpose is of great interest today. Unfortunately, despite the efforts that have been made in recent years, ability of automatic face recognition similar to the human brain is still one of the challenging research topics. For this reason, the ability of the nervous system in cognitive processes such as face recognition is considered as the future horizon of artificial intelligence systems. The methods based on feature, appearance, and hybrid tools are the most common approaches in face recognition. Among the different approaches of AI in face recognition, methods based on neural networks have received more attention due to their higher generalizability^52^. Among learning-based approaches, deep networks, especially convolutional neural networks, have found many applications in the fields of machine vision and face recognition^52–54^.

The Liquid State Machine (LSM) is a neuroscience computational model of the neural network subset that is used in real-world applications. In fact, the networks presented in this article are a type of LSM^55^. Popular face databases such as YALE and ORL was used to evaluate the performance accuracy of LSM network called pattern recognition networks based on SNN3 and SNN4.

Dataset ORL (YALE) contains 400 (165) gray images of 40 (15) different people, with 10 (11) images from each person. In this section, the performance of the pattern recognition networks based on SNN3 and SNN4 on YALE and ORL face databases was evaluated and compared with recent works. Considering that both datasets contain gray images, the background of the images has been changed to zero brightness. 80% of the data of both datasets have been used for training and the remaining 20% ​​for testing pattern recognition networks based on SNN3 and SNN4. This means that there is no commonality between the training and testing data and this helps the generalization ability of the networks. 240 training epochs were performed in learning of pattern recognition networks based on SNN3 and SNN4. Also, the performance accuracy of the pattern recognition networks based on SNN3 and SNN4 in recognizing the YALE and ORL dataset was calculated based on the average results of 15 run iterations on the test set. Table 4 shows a comparison of the performance of the proposed networks in identifying patterns of ORL and YALE compared to the best studies conducted in recent years.

**Table 4:** Comparison of the performance accuracy of SNN3 and SNN4 on YALE and ORL data set.

Accuracy on ORL dataset			Accuracy on YALE dataset		
Methods	Train/test ratios	Recognition accuracy	Methods	Train/test ratios	Recognition accuracy
G2DPCA^56^	8/2	94.79%	SNN^23^	9/2	95.7%
HNN^57^	5/5	96.2%	SPP^59^	6/5	93.13%
DSP^58^	6/4	95%	LPDP^59^	6/5	88%
RMLP^54^	4/6	96.6%	RMLP^54^	4/7	91.75%
C-TION2^54^	4/6	98.3%	C-TION2^54^	4/7	95.35%
This work (SNN4)	8/2	99.5%	This work (SNN3)	9/2	97.2%

Therefore, based on the proposed learning procedure, four pattern recognition networks called pattern recognition networks based on SNN1, SNN2, SNN3, and SNN4 for recognizing digits of EMNIST, letters of EMNIST, YALE, and ORL dataset with an accuracy of 97.8%, 93.5%, 97.2%, and 99.5%, were developed, respectively. A fundamental question has remained about the trained spiking pattern recognition networks: Which one of the four pattern recognition networks have the ability to identify an intertwined image that is obtained from the combination of two or three patterns?

In the next section, the response to this important will be examined.

## A network of the spiking pattern recognition platforms

**Figure 6:**
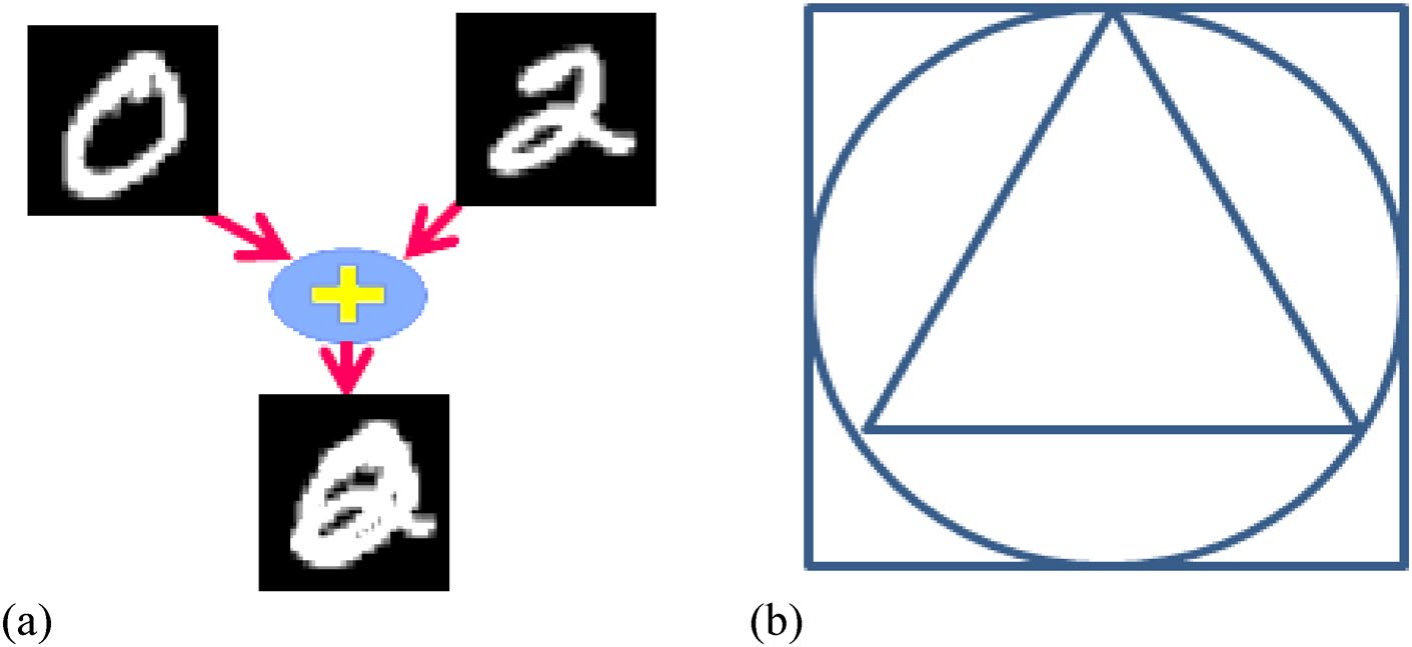
The intertwined pattern consists of square, circle, and triangle.

Spiking pattern recognition networks based on SNN1, SNN2, SNN3, and SNN4 can be used to classify digit, letters, YALE and ORL datasets, respectively. Although the reported detection accuracy in these pattern recognition networks compared to previous studies is significant, so far several pattern recognition networks have been developed to identify patterns of one dataset with an acceptable accuracy. Until now, a pattern recognition network has not provided that can detect the patterns, which obtained from combinations of two or three datasets. For example, as is evident, pattern recognition networks based on SNN1 and SNN2 are able to identify patterns of digits and letters, respectively. Therefore, if the patterns of digits and letters are combined like Fig. 6a, none of the pattern recognition networks based on SNN1 and SNN2 can detect the new patterns which called intertwined patterns. Also, Fig. 6b indicates another example of the intertwined pattern which helps clarify the importance main goal of this paper.

Man is able to recognize that Fig. 6b is a mixture of circle, square, and triangle, but how does the smart machine react to this intertwined pattern? It is evident that the machine cannot recognize this intertwined pattern, while it was trained on each of these shapes (square, circle, and triangle). This article tries to bring the recognition ability of machine closer to the cognitive processes of the nervous system as much as possible. Therefore, we emphasized on the biological background of the structure of the pattern recognition networks, which can be used to examine the functioning of the nervous system in recognizing the intertwined patterns. Also, we are looking for a solution based on biological structures that can empower the cognitive aspect of pattern recognition machines.

**Figure 7:**
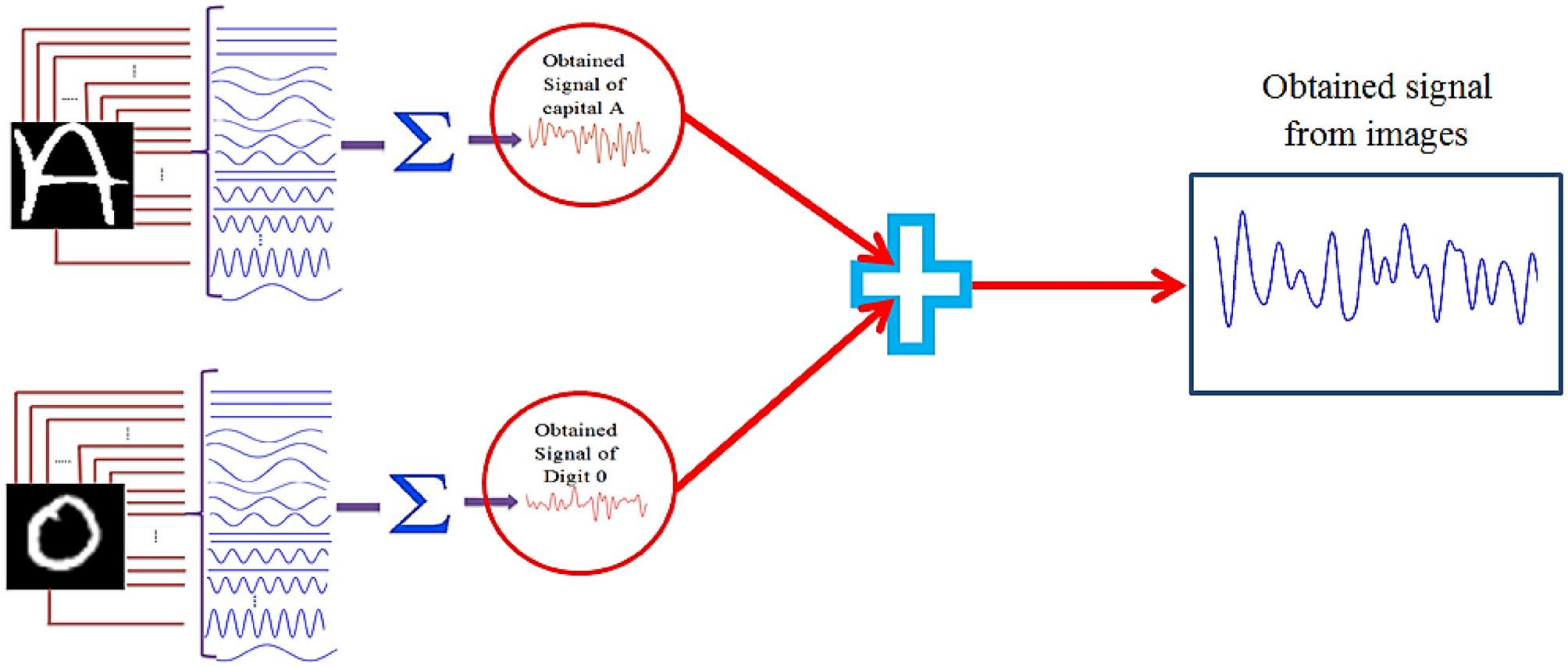
Combination of digit and letter to create the intertwined pattern of digit “0” and letter “A”. The informative signals of patterns are added to generate the new pattern that is an intertwined form of input patterns.

In order to configure a system for recognizing the intertwined patterns (patterns created by combining digits, letters, and faces), we suggested making a network of the pattern recognition platforms based on SNN1, SNN2, SNN3, and SNN4 for recognizing the complex patterns. To illustrate the concept of the intertwined pattern, Fig. 7 shows a sample of the intertwined pattern derived from the combination of the pattern of digit "0" and letter "A".

In fact, the informative signal that comes from the "intertwined" image is the same as the informative signal of Fig. 7. To clarify this subject, the informative signal of Fig. 7 is calculated as follows:

Informative signal of Fig. 7 = Informative signal of “A” from letters of EMNIST dataset + Informative signal of “Digit 0” from digits of EMNIST dataset.

Given that the size of the digits and letters is 128*128, the following relation was obtained.

16 $$ \begin{aligned} & {\text{Informative signal of}} ^{\prime\prime}A^{\prime\prime} {\text{from letters of EMNIST dataset}} \\ & \quad = \mathop \sum \limits_{i = 1}^{128} \mathop \sum \limits_{j = 1}^{128} pixel\,britnes_{ij} {\text{sin}}\left( {j*\frac{{F_{max} }}{128}*t + i*\frac{360}{{128}}} \right) \\ \end{aligned} $$

17 $$ \begin{aligned} & {\text{Informative signal of}} ^{\prime\prime}0^{\prime\prime}{\text{from digits of EMNIST dataset}} \\ & \quad = \mathop \sum \limits_{i = 1}^{128} \mathop \sum \limits_{j = 1}^{128} pixel\,britnes_{ij} {\text{sin}}\left( {j*\frac{{F_{max} }}{128}*t + i*\frac{360}{{128}}} \right) \\ \end{aligned} $$

Therefore

18 $$ \begin{aligned} {\text{Informative \, signal \, of \, Fig}}. \,{7} & = \mathop \sum \limits_{i = 1}^{128} \mathop \sum \limits_{j = 1}^{128} pixel britnes_{ij} {\text{sin}}\left( {j*\frac{{F_{max} }}{128}*t + i*\frac{360}{{128}}} \right) \\ & \quad + \mathop \sum \limits_{i = 1}^{128} \mathop \sum \limits_{j = 1}^{128} pixel britnes_{ij} {\text{sin}}\left( {j*\frac{{F_{max} }}{128}*t + i*\frac{360}{{128}}} \right) \\ \end{aligned} $$

According to Eq. (18), based on the size of the dataset images, patterns of each dataset contain different sets of frequency and phase. In fact, because the dimensions of the patterns of three datasets (digits and letters (128*128), ORL (112*92), YALE (320*243)) are different, patterns of each dataset contain sine signals with the different set of the phase and frequency. So, although the proposed conversion is nonlinear, due to the distinction of the range of phase changes and the frequency of sine signals of each dataset, there is also the possibility of converting the informative signal to the intertwined pattern.

**Figure 8:**
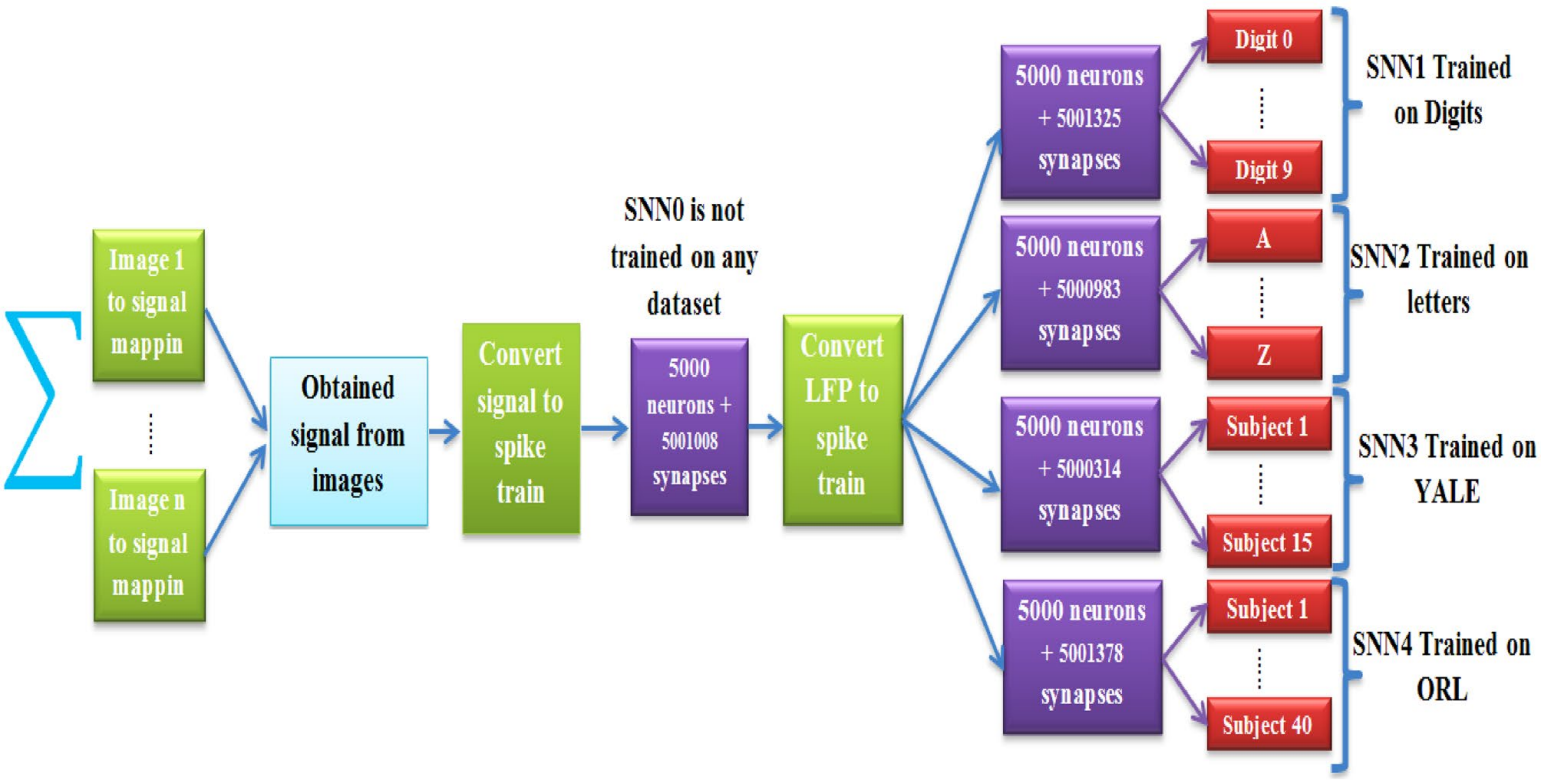
The proposed system configuration for identifying the intertwined patterns, which is based on the interaction of four spiking pattern recognition platforms of sections "Architecture and learning mechanism of the pattern recognition network based on SNN1 (digit recognition)" and "Pattern recognition networks based on SNN2, SNN3, and SNN4".

The main purpose of this paper is to provide a structure that can identify intertwined patterns, which have been generated by combining different patterns of EMNIST, YALE, and ORL datasets. Given that each of the pattern recognition networks in the previous section are capable of detecting one dataset, the network of these pattern recognition platforms enables us to identify the intertwined patterns. Therefore, the system configuration of Fig. 8 was proposed to make a network of pattern recognition platforms based on SNN1, SNN2, SNN3, and SNN4. As shown in Fig. 8, the interaction has been established between five spiking networks (SNN0,…, SNN4) to construct a platform for recognizing the intertwined patterns, for which SNN1, SNN2, SNN3, and SNN4 were trained on digits, letters, YALE, and ORL, respectively, and SNN0 is an intermediary network. It should be noted that SNN0 has the same structure as other spiking networks (SNN1,…,SNN4), with the difference that it has not been trained on any dataset, and it only plays the role of an interface between the inputs intertwined patterns and the four pattern recognition networks.

Based on the theory of “communication through coherence (CTC)”^60^, establishing the effective connection between (SNN0-SNN1) or (SNN0-SNN2) or (SNN0-SNN3) or (SNN0-SNN4) requires the fulfillment of an important condition. Scientific studies^61^ have proposed that interactions among neuronal groups are affected by synchronizations. Therefore, this important point should be considered in the effective connection between spiking networks in Fig. 8. The quantitative measure was needed to compute the level of synchrony between spiking networks over the simulation. Paul and his colleagues^62^ introduced the quantitative measurement criterion to compute the increase or decrease level of synchrony between neural populations which is used in experiential and modeling studies. The following mathematical relation was developed as synchrony measure^63,64^:

19 $$ {\Re }\left( \emptyset \right)\exp \left( {2\pi i\Phi \left( \emptyset \right)} \right) = \left( \frac{1}{n} \right)\mathop \sum \limits_{j = 1}^{n} exp\left( {2\pi i\Phi_{j} } \right) $$

where the neuron’s number, criteria of phase synchronization and mean phase are denoted by ℜ, $$\Phi$$, and $$n$$, respectively. The variance of vector $$ z$$ when $$z\left( \emptyset \right) = {\text{exp}}\left( {2\pi i\emptyset } \right)$$ is defined as follows:

20 $$ Var\left( {z\left( \emptyset \right)} \right) = \left( \frac{1}{n} \right)\mathop \sum \limits_{i = 1}^{n} \left| {z_{i} \left( \emptyset \right) - \overline{z}\left( \emptyset \right)} \right|^{2} ,\, \overline{z}\left( \emptyset \right) = \left( \frac{1}{n} \right)\mathop \sum \limits_{i = 1}^{n} z_{i} \left( \emptyset \right) $$

Based on Eqs. (19, 20), the following formula can be obtained.

21 $$ {\Re }^{2} \left( \emptyset \right) = 1 - var\left( {z\left( \emptyset \right)} \right) $$

The criterion ℜ as synchrony measure is defined in the range of 0 to 1 which the complete synchronization and de-synchronization is shown with ℜ = 1 and ℜ = 0, respectively. Based on the firing time of each neuron (spike times), the phase vector ($$\emptyset = \left( {\emptyset_{1} , \ldots ,\emptyset_{n} } \right)$$) of each neuron can be calculated. If we denote the $$k^{th}$$ spike time of neuron *i* and firing times of neuron in the interval $$[t_{i,k} ,t_{i,k + 1} )$$ with $$t_{i,k}$$ and $$T\left( {i,k} \right)$$, respectively, the time vector $$T\left( {i,k} \right)$$ can be converted to the phase vector $$Ph\left( {i,k} \right)$$ as follows^65^:

22 $$ Ph\left( {i,k} \right) = \left[ {T\left( {i,k} \right) - t_{i,k} } \right]/\left[ {t_{i,k + 1} - t_{i,k} } \right] $$

Finally, synchrony measure ℜ can be calculated based on the phase vector $Ph\left( {i,k} \right)$. It is worth noting that the synchronicity criterion ℜ must be calculated between the acceptable number of neurons to be valid, which has been proven in experimental study^62^ that the selection of 10 neurons out of 100 neurons is acceptable. Thus, we computed ℜ using 500 common neurons between SNN0 and spiking pattern recognition platforms based on SNN1, SNN2, SNN3, and SNN4.

The goal in this section is to identify the intertwined patterns by making a network of the pattern recognition platforms. The training process of pattern recognition networks based on SNN1, SNN1, SNN3, and SNN4 was done independently and prior to the making a network of them in recognizing the intertwined patterns. In Sects. 2, 3, the pattern recognition platforms based on SNN1, SNN2, SNN3, and SNN4 were trained on digits, letters, YALE faces and ORL dataset, respectively. After training process, pattern recognition platforms based on SNN1, SNN2, SNN3, and SNN4 can recognize digits, letters, YALE faces and ORL dataset with an accuracy of 97.8%, 93.5%, 97.2%, and 99.5%, respectively. What has not been addressed so far is that each pattern recognition network cannot identify its trained pattern while it has combined with patterns of other datasets. Finding a solution to this challenge is an evolution in pattern recognition systems. The synchrony measure ℜ is introduced as a diagnostic tool. System configuration of Fig. 8 using the concept of “communication through coherence” was proposed in response to the challenge of recognizing the intertwined patterns. The SNN0 acts as the interface between input intertwined pattern and the pattern recognition platforms, so that the synchronization metric of more than 0.8 determines that input consists of which combination of datasets. This means that synchrony measure ℜ > 0.8 between SNN0 and SNN1 clarify that one component of the input intertwined pattern is from digits and the maximum firing activity of the classifying neurons of pattern recognition network based on SNN1 determined which digits is the component of the input intertwined pattern. There are similar situations for the synchrony measure ℜ > 0.8 between (SNN0 and SNN2) or (SNN0 and SNN3) or (SNN0 and SNN4). Therefore, the synchrony measure ℜ is a tool for detecting components of the intertwined patterns. In the following, the main mechanism of recognizing the intertwined patterns is discussed.

According to Fig. 8, the spike train proportional to the obtained signal of input intertwined pattern excites the SNN0 and the collective behavior of neurons of SNN0 was recorded in the form of the local field potential (LFP). LFP is a general standard to evaluate the collective behavior of neurons, but the problem is that the exact relation of the LFP with the neuron variables is not fully obvious. Different computational approaches have been developed to calculate the LFP in computational models^66–68^. Some studies have computed LFP as the extracellular potential of the dendritic branches of the compartmental neuronal model^66–68^. In this paper, an analogous approach was selected with the simpler structure, in which the structure of SNNs is not assumed as a precise model of the nervous system. Therefore, LFP was considered as the sum of the absolute value of AMPA and GABA currents (|IA| +|IG|)^14^. Finally, the spike train proportional to the recoded LFP of neurons of SNN0 excites SNN1, SNN2, SNN3, and SNN4. Using an exhaustive search algorithm, we concluded that if the synchronization criterion ℜ of 500 common neurons between (SNN0-SNN1) or (SNN0-SNN2) or (SNN0-SNN3) or (SNN0-SNN4) be higher than 0.8, these two networks have strong mutual interaction. As an example, in the case that the intertwined pattern is generated by the combination of the digit “0” and letter “A”, synchronization criterion ℜ between (SNN0-SNN1) & (SNN0-SNN2) is higher than 0.8 because SNN1 was trained on digits and SNN2 was trained on letters and the spike activity of neurons of SNN0 is more synchronized with neurons of SNN1 and SNN2. Given that each of the pattern recognition networks based on SNN1, SNN2, SNN3, SNN4 have been trained on the specific dataset, the synchronization of the SNN0 with each of four networks is the first step in identifying that the input intertwined pattern consists of the combination of what datasets.

Table 5 summarizes the synchronization criterion ℜ between (SNN0-SNN1), (SNN0-SNN2), (SNN0-SNN3), and (SNN0-SNN4) in the case where the input of the proposed structure in Fig. 8 was stimulated with different intertwined patterns.

**Table 5:** The synchronization criterion ℜ when the input of Fig. 8 was excited with the different intertwined patterns.

The intertwined pattern generated by the combination of			ℜ between (SNN0-SNN1)	ℜ between (SNN0-SNN2)	ℜ between (SNN0-SNN3)	ℜ between (SNN0-SNN4)
Digit “2”	Case 24 of ORL	–	0.84	0.33	0.59	0.81
Digit “5”	Case 6 of YALE	–	0.86	0.4	0.91	0.6
Letter “D”	Case 13 of ORL	–	0.63	0.9	0.47	0.87
Letter “E”	Case 2 of YALE	–	0.49	0.85	0.89	0.51
Case 19 of ORL	Case 8 of YALE	–	0.57	0.62	0.83	0.86
Digit “7”	Letter “Z”	Case 36 of ORL	0.84	0.9	0.67	0.88
Digit “8”	Letter “M”	Case 7 of YALE	0.91	0.81	0.87	0.71

As shown in Table 5, the SNN0 acts as the interface between input intertwined pattern and the pattern recognition platforms, so that the synchronization metric of more than 0.8 determines which combination of dataset create the input. This means that if the input intertwined pattern is the combination of Digit “2” and subject 24 of ORL, then the interface network SNN0 with the synchronization metric higher than 0.8 is synchronized with the SNN1 and SNN4, which were trained on digits and ORL faces, respectively. Therefore, synchronization between SNN0 with each of the pattern recognition platforms determines which datasets have been combined to form the input intertwined pattern. Finally, the classifying neurons of each pattern recognition platforms based on SNN1, SNN2, SNN3, and SNN4 that was synchronized with the SNN0 determine which patterns are combined to create the input intertwined pattern. For example, when the input intertwined pattern is the combination of Digit "0" and letter "A", the classifying neuron "0" in the pattern recognition network based on SNN1 and the classifying neuron "A" in the pattern recognition network based on SNN2 has the highest rate of spike activity compared to the other classifying neurons of these pattern recognition networks. Table 6 indicates the mean of the correct recognition rate of the system configuration of Fig. 8 in classifying the input intertwined pattern. The result of Table 6 illustrates the ability of the system configuration of Fig. 8 in recognizing the intertwined patterns. As far as we can tell, these results are illustrated for the first time, in which a network of spiking pattern recognition platforms in the form of Fig. 8 can be used in recognizing the intertwined patterns.

**Table 6:** The rate of correct recognition of system configuration Fig. 8 in classifying the intertwined patterns, which were obtained from the combination of patterns of two or three datasets.

The datasets that are used to generate the intertwined pattern			The number of created intertwined patterns	The recognition rate of digits among the intertwined patterns	The recognition rate of letter among the intertwined patterns	The recognition rate of YALE faces among the intertwined patterns	The recognition rate of ORL faces among the intertwined patterns
Digit of EMNIST	ORL faces	–	210 digit*80 ORL = 16,800	95.47%	–	–	95.97%
Digit of EMNIST	YALE faces	–	210 digit*30 YALE = 6300	93.65%	–	91.44%	–
Letters of EMNIST	ORL faces	–	546 letter*80 ORL = 43,680	–	90.69%	–	96.33%
Letters of EMNIST	YALE faces	–	546 letter *30 YALE = 16,380	–	90.09%	91.6%	–
YALE faces	ORL faces	–	80 YALE*30 ORL = 2400	–	–	79.58%	82.87%
Digit of EMNIST	Letters of EMNIST	ORL faces	9,172,800	92.35%	86.28%	–	90.74%
Digit of EMNIST	Letters of EMNIST	YALE faces	3,439,800	90.4%	85.02%	89.81%	–

The first three columns of Table 6 represent the datasets used to create the intertwined patterns. Based on the number of the considered test samples of each dataset, the number of intertwined patterns are reported in the fourth column. The intertwined patterns are a combination of two or three patterns. The remarkable novelty in this article is that we can recognize that the intertwined patterns have been constructed of what patterns. The four last columns in Table 6 show the correct detection rate of the proposed network (Fig. 8) in identifying the created intertwined patterns. For example, the first row of Table 6 shows that the proposed network of Fig. 8 is able to accurately identify the digits of 16,800 intertwined patterns in 95.47% of cases and correctly classify facial patterns in 95.97% of cases.

Network performance analysis of Table 6 indicates that the intertwined patterns can be classified accurately by the maximum firing activity of the classifying neurons of the proposed configuration. The proposed network of pattern recognition platforms can be a valuable candidate in the modeling of cognitive ability of the nervous system in intelligent machines.

Pattern recognition networks based on SNN1, SNN2, SNN3, SNN4, respectively need 4, 4, 240, and 240 training epochs to reach the reported accuracies. Actually, in the first training epoch, the classification neurons of the pattern recognition networks randomly respond to the input patterns and classification accuracy is very low. This means that pattern recognition networks are able to accurately classify digits, letters and faces when the training process is complete. But in the case of the intertwined pattern recognition network, SNN0 provides the possibility to recognize intertwined patterns based on the separation of frequency components. In fact, in this network, the input coding plays a fundamental role in the classification of the complex patterns.

## Conclusion

This paper designs pattern recognition networks and spatial-STDP learning approach based on the structure and characteristics of learning in the nervous system. Past studies have introduced the STDP learning method in the training of spiking neural networks as un-supervised learning based on biological principles. On the other hand, some studies have shown that LTP and LTD are the two dominant behavioral models in the information storage mechanism, memory, synaptic plasticity, and learning in the nervous system^69,70^. Accordingly, in this article, we present the spatial STDP learning approach that spatial LTP and LTD by affecting the release of AMPA and GABA neurotransmitters regulates synaptic weights to learn patterns. In this regard, four spiking pattern recognition networks were designed based on the computational model of cortical (SNN1, SNN2, SNN3, and SNN4). First, an attempt was made to train these networks on EMNIST, YALE and ORL faces datasets. After the completion of training, the pattern recognition networks based on SNN1, SNN2, SNN3, and SNN4 can classify digits of EMNIST, letters of EMNIST, YALE faces and the ORL test-set with an high accuracy of 97.8%, 93.5%, 97.2%, and 99.5% which is more accurate compared to previous related works. Each of the pattern recognition networks is able to identify the patterns of one dataset and cannot recognize the intertwined patterns. Until now, no network has been provided to recognize the intertwined patterns. This means, there is still no pattern recognition network that has the ability to integrate the capability of multiple pattern recognition networks in one system configuration. The reported results in this paper indicate that the proposed system for classifying the intertwined patterns has the ability to separate the components of the input intertwined pattern with high precision.

## Data Availability

Data would be available through corresponding author with reasonable request.
